# Characterizing the Deformation of the Polydimethylsiloxane (PDMS) Membrane for Microfluidic System through Image Processing †

**DOI:** 10.3390/mi7050092

**Published:** 2016-05-16

**Authors:** Xiang Qian, Wenhui Zhang, Cheng Peng, Xingyang Liu, Quan Yu, Kai Ni, Xiaohao Wang

**Affiliations:** 1 Graduate School at Shenzhen, Tsinghua University, Shenzhen 518055, China; zhangwh13@mails.tsinghua.edu.cn (W.Z.); liuxingyang13@mails.tsinghua.edu.cn (X.L.); yu.quan@sz.tsinghua.edu.cn (Q.Y.); ni.kai@sz.tsinghua.edu.cn (K.N.); wang.xiaohao@sz.tsinghua.edu.cn (X.W.); 2 Department of Biology, South University of Science and Technology of China, Shenzhen 518055, China; pengc@sustc.edu.cn; 3 The State Key Laboratory of Precision Measurement Technology and Instruments, Tsinghua University, Beijing 100084, China; 4 The Research Institute of Tsinghua University in Shenzhen, Shenzhen 518057, China

**Keywords:** microfluidic, PDMS membrane, level set, image processing, capacitance

## Abstract

Polydimethylsiloxane (PDMS) membranes have been widely used in the microfluidic community to achieve various functions such as control, sensing, filter, *etc.* In this paper, an experimental process was proposed to directly characterize the deformation of the on-chip PDMS membrane at large deformation based on the image processing method. High precision pressures were applied on the surface of the PDMS membrane with fixed edges and a series deformation of the PDMS membrane were captured by the imaging system. The Chan and Vese (CV) level set method was applied to segment the images of the deformed membrane. The volumes wrapped by the deformed membranes were obtained, and pressure-volumes relationships of the PDMS membranes with different geometry parameters were also calculated. Then the membrane capacitance can be derived by differentiating the curve of pressure-volumes. In addition, the theoretical estimation of the capacitance of the PDMS membrane at large deformation was also obtained through finite element simulation (FEM), which was in good agreement with the experimental results. These results are expected to be significant for designing and on-chip measuring of such PDMS membrane based microfluidic components in our future work.

## 1. Introduction

In the microfluidic and micro-machine community, polydimethylsiloxane (PDMS) based components have many advantages, such as good optical transparency, excellent insulation, hyper elasticity, biocompatibility, and easy to fabricate the low-cost devices through soft lithography, *etc.* Thus, the PDMS membrane structures were often designed to achieve specific functions in the microfluidic systems, for example, the microfluidic filters [1], the microfluidic pumps [2], the microfluidic digital-to-analogue converters (DACs) [3] and the microfluidic sensors [4]. Mechanical properties of such PDMS membrane structures may affect the response of these microfluidic devices.

Several studies about the deformation of the PDMS membrane have been extensively reported in the literature [5,6,7,8,9,10,11,12], most of which assumed the contours of the deformed PDMS membrane to be spherical caps or other determinate shapes, in other words, the membrane capacitance defined as the volumes change caused by the unit pressure change was regarded as a constant. For example, the close-form analytic expression of the hydraulic capacitance of a PDMS based microfluidic channel can be derived related to the radius, the thickness, the elastic modulus and the Poisson’s ratio of the PDMS membrane [13]. Such a model is only compatible with small deformation and not accurate when the PDMS membranes encounter large deformation in real microfluidic systems. In a significant work for designing the microfluidic filters reported previously [1], a thin layer of gold was coated on the PDMS membrane to greatly increase the effective elastic modulus of the membrane by several order of magnitudes, which finally caused only small deformations. From the classical theory point of view, the deformation of the PDMS membrane can be modeled by several nonlinear partial differential equations, which are difficult to solve without simplifications. In this regard, early and recent efforts including the thin-plate theory for large elastic deformations [14] and the large deformation isotropic elasticity theory [15], both relate to the strain energy equation, were proposed to solve the large deformation model. The non-linear pressure-flow relationship of a PDMS membrane based microfluidic diode was also studied through a simplified close-form analytical solution [16].

On the other hand, experimentally characterizing the deformation of the PDMS membrane and estimating the membrane capacitance according to different applied pressures, PDMS curing conditions and membrane geometries gave an alternative way to model and design such micro-valves, micro-pumps, micro-filters and micro-sensors in the microfluidic systems. In the recent report by Lau *et al.* [17], the dynamic of the PDMS based micro-valve was investigated through an indirect image processing method, that is to say, when embed into an integrated microfluidic circuits, the closure time and the releasing time of the micro-valve were estimated through the intensity of the red dye beneath the micro-valve firstly, and then the capacitance of the PDMS membrane was further estimated. In this paper, we proposed a direct image processing method to calculate the PDMS membrane capacitance. Based on a custom made testing component of the PDMS membrane, high precision pressure was applied on the surfaces of the round PDMS membrane with fixed edges and the deformation of the PDMS membrane were captured by the microscopy imaging system. Image segmentation and curve fitting methods were applied to calculate the volumes wrapped by the PDMS membrane to obtain the pressure-volumes relationship; and the membrane capacitance can be obtained by differentiating the curve of the pressure-volumes relationship. Although this paper was an illustration workflow using a custom made testing component, the proposed method can no doubt be used for on-chip measurement of the hydraulic capacitance of the PDMS membrane based components in real microfluidic system before final packaging, without knowing the geometry parameters and the material parameters of such PDMS membrane. That is to say, a single layer of the microfluidic channel is firstly bonded to a working PDMS membrane and tested through the proposed method, and the other microfluidic layers are then bonded to make a final package. Furthermore, based on the analysis of the experimental results and the finite element simulations (FEM), the empirical formula of the PDMS membrane capacitance can be obtained, which was supposed to be significant for designing such PDMS membrane based components through an electric circuit analogy approach [13,18].

## 2. Theoretical Analysis

**Figure 1:**
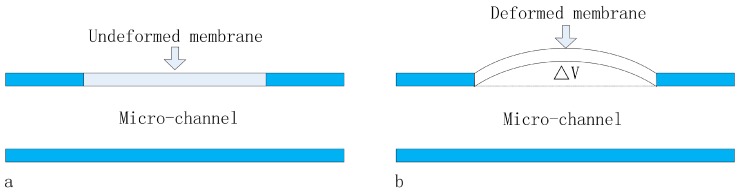
The cross section of a membrane-based microfluidic capacitor: (**a**) an uncharged capacitor and (**b**) a charged capacitor.

The hydraulic capacitance (*C*) is defined as the volumes change per unit pressure variation as follows:

(1) $$Q=\frac{dV}{dt}=\frac{dV}{dP}\frac{dP}{dt}=C\;\frac{dP}{dt}$$

where *Q*, *V*, *P* represent the mean flow rate, the volumes change and the applied pressure respectively, which are equivalent to the electric current (*I*) the electric current (*Q*) and the electric current (*V*) in the electric circuit; and the hydraulic capacitance is equivalent to the electric capacitor [13]. The hydraulic capacitance should not be ignored when (i) the structure of the system is flexible; (ii) bubbles exist inside the microfluidic system; or (iii) the fluid inside the system is compressible. Generally, bubbles in the micro-channel are released, the liquids are supposed to be incompressible and the deformation of the micro-channel thick wall is neglected, and the thin layer of the PDMS membrane is the only factor that must be considered to affect the hydraulic capacitance in a microfluidic system, in other words, the capacitance of the PDMS membrane is just the hydraulic capacitance in the microfluidic channel, as shown in Figure 1.

This paper considers the round PDMS membrane with fixed edge; thus, a simple analytical analysis was performed to compare with the experimental results considering such conditions. However, the PDMS membrane with other symmetrical geometries is also suitable for the proposed experimental method. The corresponding capacitance of the round PDMS membrane varies in the different deformation models. The small deformation model follows the theory of Poisson’s small deflections of a circular plate, and the equilibrium equation is,

(2) $$\frac{1}{R}\frac{d}{dR}\left\{R\frac{d}{dR}\left[\frac{1}{R}\frac{d}{dR}\left(R\frac{dw}{dR}\right)\right]\right\}=\frac{P}{D}\;D=\frac{Eh^{3}}{12\left(1-\mu^{2}\right)}$$

where *R* is the radius of the membrane *w* is the deflection of the membrane, *P* is the applied pressure, *μ* is Poisson’s ration, *E* is Young’s modulus of PDMS and *h* is the thickness of the membrane. The analytical expression of the capacitance can be acquired from the above equation, as follows [13]:

(3) $$C=\frac{\pi \left(1-\mu^{2}\right)R^{6}}{16Eh^{3}}$$

This analytic expression is only limited to *h/R* ≪ 1 and *w/h* ≪ 1.

**Figure 2:**
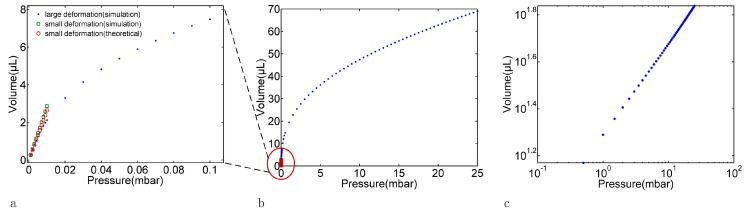
The pressure-volumes relationship of a deformed membrane based on the large deformation model and the small deformation model, (**a**) the magnifying view for the small deformation region; (**b**) the total view for the large deformation model; (**c**) the logarithmic view for the large deformation model. The uniform gas pressure was applied on the membrane, and the volume change was defined as the space wrapped by the outer boundary of the deformed membrane. The blue dot is the result of finite element simulation (FEM) based on the large deformation model, the green square is the result of FEM based on the small deformation model and the red circle is the result of small deformation based on the Equation (3).

Large deformation model follows the theory of Karman’s large deflection for a circular plate, and the equations are as follows [19]:

- Equilibrium equation
  (4) $$\frac{d}{dR}\frac{1}{R}\frac{d}{dR}R\frac{dw}{dR}-N_{R}\frac{dw}{dR}=\frac{1}{R}\int_{0}^{R}PRdR$$
- Compatibility equation
  (5) $$R\frac{d}{dR}\left[\frac{1}{R}\frac{d}{dR}\left(R^{2}N_{R}\right)\right]+\frac{Eh}{2}{\left(\frac{dw}{dR}\right)}^{2}=0$$

where *N_R_* is the radial force. The Karman equations are difficult to solve analytically without simplifications. In this paper, we solve the Equations (4) and (5) numerically using FEM method. Different pressure-volumes relationship based on the large deformation model and small deformation model can be observed as shown in Figure 2. The uniform gas pressure was applied on the membrane, and the volumes change was defined as the space wrapped by the outer boundary of the deformed membrane. As it can be seen in Figure 2a (a magnifying view for the small deformation region, where the applied pressure was under 5 × 10^−3^ mbar and the volumes change was under 1.5 μL), the theoretical values (red circle) and the FEM values (green square for small deformation model and the blue dot for large deformation model) are in good agreement to a linear fitting model, that is to say the membrane capacitance defined as the slope of such pressure-volumes curve can be treated as a constant. However, in the region where the applied pressure was above 5 × 10^−3^ mbar and the volumes change was above 1.5 μL), as so called large deformation region, the volumes change curve of the large deformation model was far from that of the small deformation model, as shown in Figure 2b. Such a pressure-volumes relationship was supposed to fit linearly on a logarithmic scale (as shown in Figure 2c), such that

(6) LogV=mLogP+n

where *m* and *n* are fitting parameters. Thus, the pressure-volumes relationship can be expressed in an exponential form

(7) $$V=NP^{m},\;N=10^{n}$$

and the corresponding membrane capacitance is

(8) $$C=\frac{dV}{dP}=mNP^{m-1}$$

As a conclusion, from the theoretical point of view, the large deformation model can be solved numerically using FEM method and the hydraulic capacitance of a PDMS membrane based component can be obtained using Equation (8). As shown in Equations (4) and (5), the geometry parameters such as the radius *R* and the thickness *h*, and the material parameters such as the Young’s modulus *E* and the Poisson’s ratio *μ* should be known before calculation. Such simulation can be used to derive an empirical expression for a group of given parameters that may contribute to designing these PDMS membrane based components. However, for the real microfluidic systems, these parameters may vary according to the fabrication process; an experimental process without any known parameters was supposed to be more accurate and simple. In the next section of this paper, an experimental setup was designed and an image processing method was proposed to obtain the membrane capacitance. The proposed process can be used for on-chip measurement of hydraulic capacitance of the real microfluidic components before final package.

## 3. Materials and Methods

### 3.1. Experimental Setup

RTV615 PDMS elastomer pre-polymer and curing agent were purchased from Momentive (Waterford, New York, NY, USA). The pressure source was a nitrogen bottle, which was regulated by a pneumatic pressure controller (MFCS, Fluigent, Paris, France) to produce high precision pressure to apply on the surfaces of the round PDMS membrane. This pneumatic pressure controller allowed almost non-fluctuating flow, which was essential to maintain the stability of the deformed membrane. The uniform gas pressures exerted on the membrane were from 0 to 25 mbar and the pressure step was 0.5 mbar. A high-speed camera (ORCA-flash, Hamamatsu, Shizuoka, Japan) mounted on an inverted optical microscope (Eclipse TE 2000-U, Nikon, Tokyo, Japan) was used to get the image of the deformed membrane.

**Figure 3:**
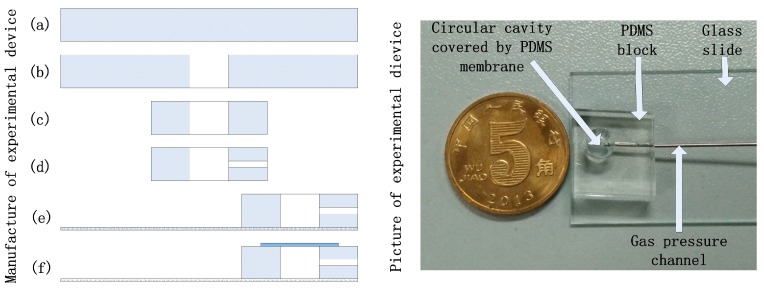
(**Left**) Flow charts of the fabrication process for the polydimethylsiloxane (PDMS) membrane testing chamber: (**a**) Fabricate the PDMS pie with parallel surfaces; (**b**) Punch a round hole in the PDMS pie; (**c**) Cut the PDMS pie into a small block with a round hole; (**d**) Punch a side hole in small PDMS block used as the uniform pressure channel; (**e**) Bond the PDMS block and microscope slide together by plasma treatment to ensure the seal; (**f**) Bond the PDMS membrane and PDMS block together, covering the round hole and obtaining a circular membrane, or acts as an unfinished micro-chip; (**Right**) Picture of the final experimental device.

**Figure 4:**
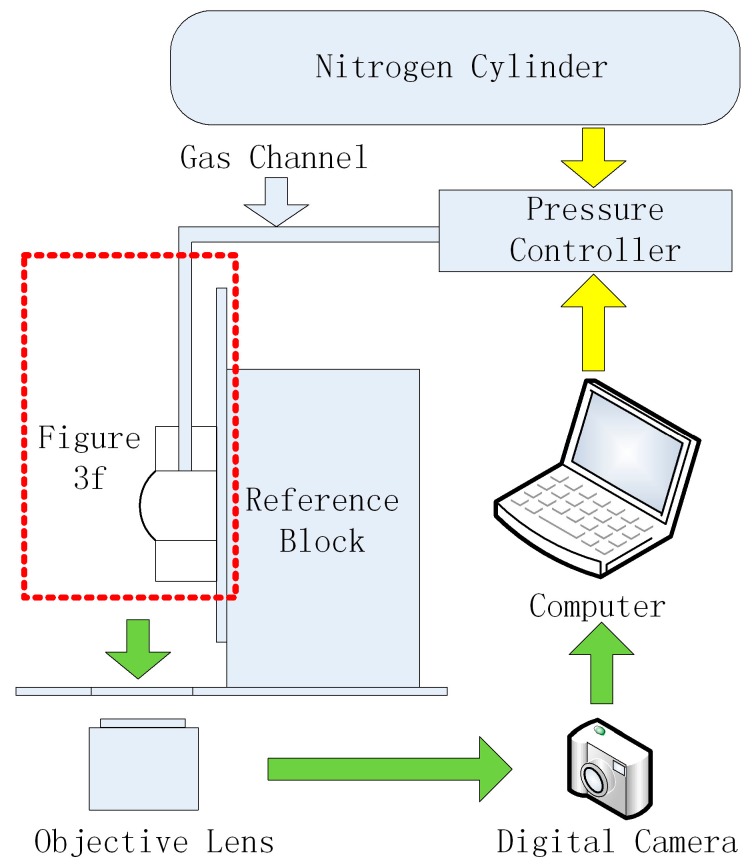
The experimental setup for PDMS membrane testing. The nitrogen bottle acted as a source of the gas pressure, the pressure controller accepted orders from the computer to control its output pressure and connected with the testing device in Figure 3 by gas channel, the testing device was fixed on the reference block to keep vertical to the stage, the digital camera captured the images of the deflected membrane through the objective lens.

The custom made PDMS membrane testing chamber in this paper was fabricated as follows, as shown in Figure 3. A small PDMS block was fabricated by pouring the mixture of PDMS pre-polymer and curing agent (10:1 *w*/*w*) into a petri dish, keeping the thickness about 5 mm, putting the petri dish on a horizontal table at room temperature, and baking the PDMS properly to increase its hardness to form a PDMS pie after solidification with parallel surfaces (Figure 3a). Holes with different diameters were punched on the PDMS pie (Figure 3b), the PDMS pie was cut into small blocks containing round holes (Figure 3c). A side channel in the small PDMS block was punched to communicate with the round hole, which can be used as the uniform pressure channel (Figure 3d). The small PDMS block and the microscope slide was bonded together through oxygen plasma (Figure 3e). The PDMS membrane was fabricated through spin-coating that the mixture of PDMS pre-polymer and curing agent was poured onto a polished silicon wafer; different mixing ratio of the pre-polymer and curing agent, and the rotational speed and time for spinning were adjusted to fabricate the PDMS membranes with different thickness and mechanic properties. The PDMS membrane covering wafer was then baked in an oven at 80 °C for 40 min, and finally the PDMS membrane was cut into the right size, tiled on a PMMA block without tension (was not shown in Figure 3), and bonded to the small PDMS block by oxygen plasma (Figure 3f). The PMMA block acted as the carrier of the PDMS membrane, which can reduce the preload tension of the PDMS membrane and keep the PDMS membrane more smooth. High precision pressure was applied to the chamber through the MFCS pressure controller. By adjusting a series of stable pressures, a series of corresponding deformations of the PDMS membrane was obtained by the imaging system. The experimental setup for PDMS membrane testing is shown in Figure 4.

### 3.2. Image Processing

**Figure 5:**
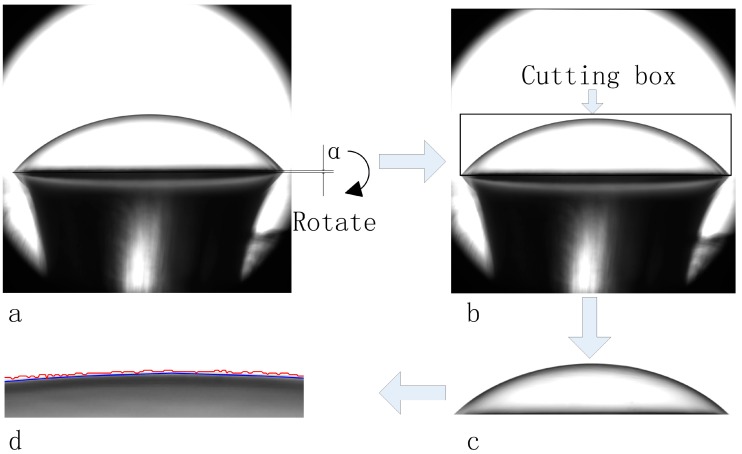
Flow chart for the proposed image processing method to obtain the outer boundary of the deformed membrane: (**a**) the original image with an image rotation angle *α*; (**b**) the result of rotating and cutting the image using a region of interest (ROI) box; (**c**) the result of cutting; (**d**) the result of segmentation by thresholding method (red line) and the level set method (blue line).

According to previous described experimental process, a series images of the deformed PDMS membrane can be obtained, Figure 5a illustrates one such microscopy image. Causing by the relative position of the testing chamber and the image system, the original microscopy images may have a certain rotation angle *α*, that need to be rotated back to keep the images’ baseline horizontality, as shown in Figure 5a. A region of interest (ROI) cutting box containing the PDMS membrane was then applied to the original image as shown in Figure 5b,c.

After preprocessing, the image segmentation methods were applied to the cutting ROI regions to obtain the outer boundary of the PDMS membrane. It seemed that directly thresholding on the image using Otsu’s method [20] was failed to obtain a smooth boundary, as shown in Figure 5d redline. Thus, the Chan and Vese (CV) level set algorithm [21] was applied to segment the PDMS membrane image. The two-phase image model with piecewise-constant intensity was assumed such that the low intensity region represented the PDMS membrane contained region and the high intensity region represented the illumination background. The fitting energy for such an image model can be represented as,

(9) $$\varepsilon =\int_{PDMS}{\left|u\left(x,y\right)-c_{PDMS}\right|}^{2}dxdy+\int_{background}{\left|u\left(x,y\right)-c_{background}\right|}^{2}dxdy$$

where *u(x, y)* is the image intensity, *C_PDMS_* is the mean intensity for the PDMS contained region and *C_background_* is the mean intensity of the background region. To segment the PDMS membrane boundary, such fitting energy should be minimized under regularization as,

(10) $$inf_{C_{PDMS}}\left\{\mu \times Length_{C_{PDMS}}+\varepsilon \right\}$$

where *C_PDMS_* represents the PDMS membrane boundary and *μ* is the regulation parameter. Such minimization problem can be solved using the level set formation, by embedding the *C_PDMS_* into the zero level of a level set function ϕ and evolution the level set function to its steady state as

(11) $$\frac{d\varphi }{dt}=\delta \left(\varphi \right)\left[\mu div\left(\frac{\nabla \varphi }{\left|\nabla \varphi \right|}\right)-{\left(u-c_{PDMS}\right)}^{2}+{\left(u-c_{background}\right)}^{2}\right]$$

where *δ*(*ϕ*) represents the smooth version of the delta function. In this paper, we adopted the original discretization scheme as in [20] and applied the additive operator splitting (AOS) method to solve the Equation (11) efficiently.

Figure 5d blue line is the result of the CV model segmentation for getting the outer boundaries of deformed membrane. It can be clearly observed that, the CV model can segment the two phase piecewise constant image (dark region as the PDMS membrane and the bright region as the background) with a smooth boundary. One thing should be additionally mentioned: the microscopy image of the PDMS membrane can be regarded as the blurred image of the “true” PDMS membrane, which was caused by the imaging system. Such blurred image can be restored by minimizing the ROF (Rudin, Osher and Fatemi) image restoration model as,

(12) $$\varepsilon =\mu \int {\left(f-g\right)}^{2}dxdy+\int \text{​}\left|\nabla g\right|dxdy$$

where *f* represents the blurred image and *g* represents the “true” image. It was suggested that, the segmentation model in Equation (10) should be identical to the ROF image restoration model in Equation (12) with thresholding [22], so that the CV model was supposed to segment the “true” boundary of the PDMS membrane. The outer boundary of the PDMS membrane was further fitting by a sixth order polynomials, so that firstly the horizontality can be further verified by the symmetry of the boundary and the secondly the volume that wrapped by the PDMS membrane can be obtained. Several parameters in the above proposed image processing algorithms were manually set in our current experiment, such as the image rotation angle, the ROI box and the regulation parameter μ; however, these parameters can be fixed in the future applications through a customized microscopy system with fixed field of view and illumination.

## 4. Result and Discussion

### 4.1. FEM Results

**Figure 6:**
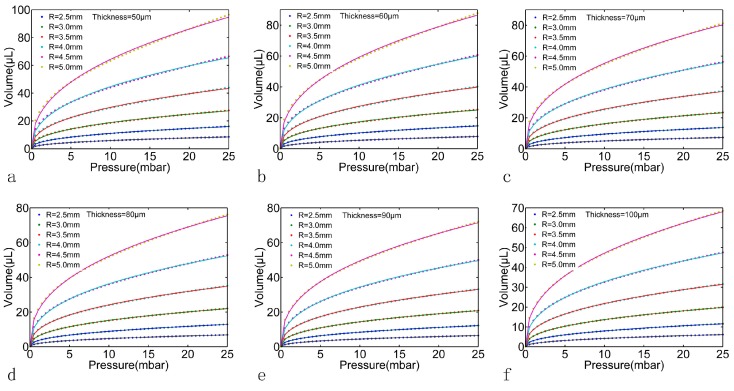
The pressure-volumes relationships of the PDMS membrane with different radius R and different thickness *h* derived from the FEM analysis. Subplot (**a**–**f**) represent the thickness of the PDMS membrane from 50 to 100 μm with 10 μm a step.

**Figure 7:**
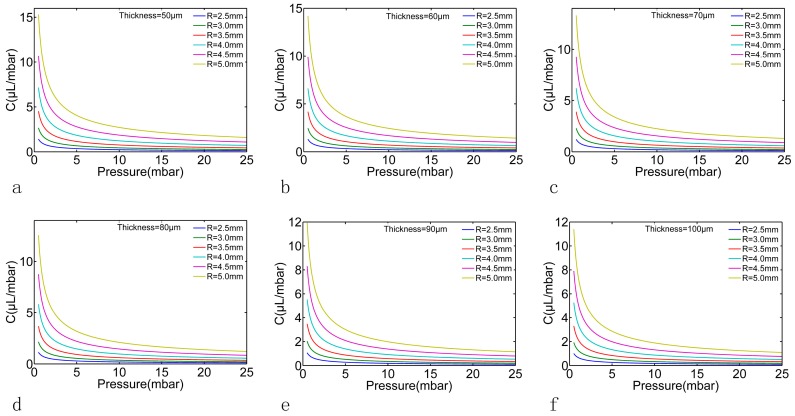
The pressure-capacitance relationships of the PDMS membrane with different radius R and different thickness *h* derived from the FEM analysis and Equation (8). Subplot (**a**–**f**) represent the thickness of the PDMS membrane from 50 to 100 μm with 10 μm a step.

In order to obtain the relationship between the geometry parameters *R*, *h* and the fitting parameters *m*, *N*, FEM models with different *R* and *h* were calculated in this paper. The *R* was set as 2.5 mm to 5 mm with 0.5 mm a step and the *h* was set as 50 μm to 100 μm with 10 μm a step. The Young’s modulus *E* was set to 8.82 × 10^5^ Pa (according to the material test by the texture analyzer CT30-10kg, Brookfield AMETEK, MA, USA), the Poisson’s ratio *μ* was set to 0.49 and the applied pressure was set to the same as that in experiment (0 mbar to 25 mbar with 0.5 mbar a step). During the material test, the applied stress was from 0–5 MPa, and the linear region was around 0.2 MPa. However, the applied pressure in our microfluidic system was below 25 mbar by a high precision-driven pressure, which is much lower than 0.2 MPa; this ensured that the membrane test took place in the linear region. The FEM results are shown in Figure 6. Each pressure-volumes curve in Figure 6 (dot plot) can be fitted using Equations (6) and (7). As shown by the solid line in Figure 6, the fitting RSD was above 99% for all curves. The fitting parameters *m* and *N* are shown in Table 1; according to these fitting parameters, the capacitance of the PDMS membrane with each geometry parameter set can be calculated using Equation (8), as shown in Figure 7.

**Figure 8:**
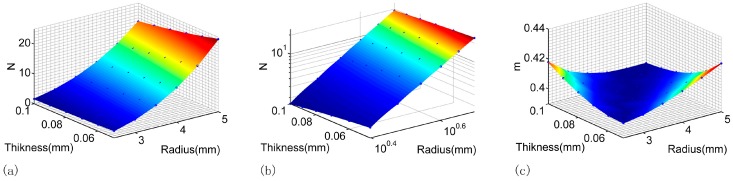
2D surface plot of (**a**) the [*R*, *h*] → *N* relationship; (**b**) the [*R*, *h*] → *m* relationship in logarithmic scale and (**c**) [*R*, *h*] → *m* relationship. The dot in each plot represents one certain FEM calculated point in Table 1, and the surface in each plot represents the fitting function.

Furthermore, the [*R*, *h*] → *m* and the [*R*, *h*] → *N* relationship in Table 1 can be plotted and fitted using 2-D functions as shown in Figure 8. The [*R*, *h*] → *N* relationship was linear fitting (*RSD* = 99.9%) in logarithmic scale as shown in Figure 8b, such that

(13) LogN=3.439logR−0.4285logh−1.561

and thus

(14) $$N=\frac{R^{3.439}}{36.3915h^{0.4285}}$$

The [*R*, *h*] → *m* was quadratic polynomial fitting (*RSD* = 97.6%) as shown in Figure 8c, such that

(15) $$m=0.4077+0.0003718R-0.1879h+0.003195R^{2}-0.3158Rh+8.596h^{2}$$

Based on the observations on Equations (13)–(15), we can firstly speculate that the *N* value was influenced by the radius R more than thickness *h* as illustrated by Equation (13); and secondly, the *m* value in Equation (15) can be approximately set to 0.41 according to the current geometry parameters that the radius *R* was in mm scale and the thickness *h* was in μm scale. Incorporating these two observations into Equation (8), we finally arrived at an empirical estimation of the capacitance of such a PDMS membrane:

(16) $$C\approx 0.41NP^{-0.59}$$

and from the above equation, it can be concluded that the overall capacitance *C* was also influenced by the radius *R* more than the thickness *h*, which is consistent with the illustration in Figure 7. Another observation can be drawn from Figure 7 and Equation (16) was that for larger applied pressure such as 20 mbar to 25 mbar or even above, the difference of the capacitance with difference geometry parameters became small, which suggests that the optimal working pressure for such PDMS membrane is under 25 mbar.

### 4.2. Experimental Results

**Figure 9:**
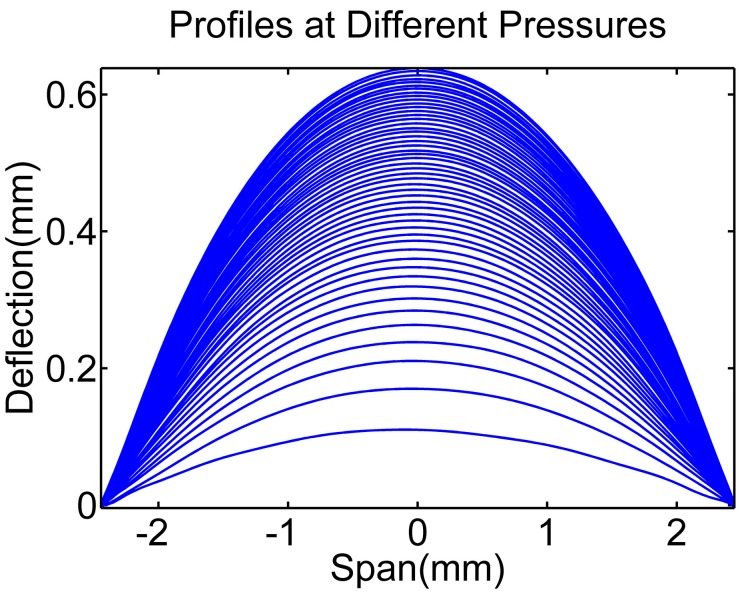
Profiles of deformed membrane in experiments, the exerted pressures are from 0 to 25 mbar with the step of 0.5 mbar.

**Figure 10:**
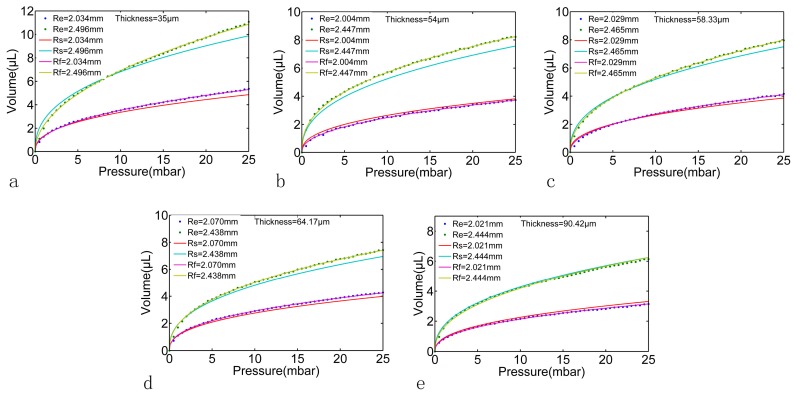
The experimental pressure-volume relationships of the PDMS membrane with different radius *R* and different thickness *h*. The “Re” dot plot indicate the results of the experiments and the “Rf” solid lines indicate the results of fitting the experimental data. The “Rs” solid line indicate the theoretical estimation using the same geometry parameters and Equations (13)–(15). Subplot (**a**–**e**) represent the measured thickness of the PDMS membrane from 35 to 90 μm.

**Figure 11:**
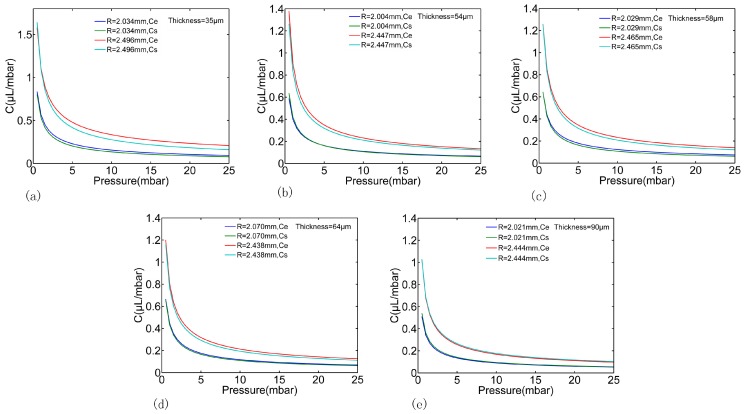
The experimental pressure-capacitance relationship of the PDMS membrane. “Ce” and “Cs” are the hydraulic capacitances obtained by experiments and theoretical estimation respectively. Subplot (**a**–**e**) represent the thickness of the PDMS membrane from 35 to 90 μm.

According to the above described experimental process, a series of the boundaries of one deformed PDMS membrane by gradually increasing pressure were obtained through the proposed image processing method, as shown in Figure 9. We tested several times for each working pressure. According to the high precision pressure driven, the microscopy image system and the proposed algorithm, we can get fairly accurate measurements that show that the difference between different tests was below 0.5%, which means a 0.5 μL difference at 100 μL. Thus, the experimental pressure-volumes relationships for the PDMS membranes with different thickness and radius can be obtained, as shown in Figure 10 “Re” dot plot. In this paper, the geometry parameters in all experiments were measured as illustrated in Figure 10 for comparison; however, these parameters can be unknown in prospective real applications. The experimental results were also fitted using Equations (6) and (7) as shown in Figure 10 “Rf” solid line. The theoretical estimation of the same geometry parameters were also calculated using Equations (13)–(15), as shown in Figure 10 “Rs” solid line. As it can be seen, the results of experiments and the results of simulations have the same tendency and are good agreement with each other, the errors of all the cases carried out in this paper were less than 8%. As a result, the experimental pressure-capacitance curve can be obtained using Equation (8) as shown in Figure 11.

Gathering the FEM results and the experimental results together, it can be concluded that the theoretical estimation of the capacitance using Equation (16) can serve as a designing reference for PDMS membrane based microfluidic components with known geometry parameters. However, for real on-chip applications, the proposed experimental process and image processing methods are expected to be more accurate and simpler without the requirement of measuring the geometry parameters and the material parameters beforehand.

## 5. Conclusions

In this paper, an experimental process was proposed to characterize the deformation of the on-chip PDMS membrane at large deformation for microfluidic systems through image processing method. The experimental platform was built to obtain the deformations of PDMS membranes using custom made testing chambers. The image processing method based on the Chan and Vese (CV) level set algorithm was applied to segment the outer boundary of the deformed PDMS membrane. FEM based theoretical estimation of the capacitance of the PDMS membrane at large deformation was obtained and was in good agreement with experimental results. These results indicated that the relationship between the volumes wrapped by the PDMS membrane and the applied pressures can fit in the logarithmic scale in the so called large deformation region and the capacitance of the PDMS membrane can be obtained through differentiating the pressure-volumes curve. The proposed process can be used for direct on-chip measurement of the hydraulic capacitance of the PDMS membrane based microfluidic components accurately and easily. Furthermore, the FEM based theoretical estimation of the capacitance is expected to be significant for designing the PDMS membrane based microfluidic components in our future work.

## Figures and Tables

**Table 1:** The value of the parameters *m* and *N* with different *R* and different thickness *h*.

*h* (μm)	*R* (mm)	N	m	*R* (mm)	N	m	*R* (mm)	N	m
50	2.5	2.3479	0.39991	3	4.38163	0.40200	3.5	7.3833	0.40593
60		2.1567	0.40036		4.05922	0.3997		6.88002	0.40156
70		1.9934	0.40281		3.78201	0.39986		6.45017	0.3996
80		1.8493	0.40677		3.53845	0.40148		6.06283	0.39992
90		1.7207	0.41172		3.31882	0.40452		5.73033	0.40065
100		1.6020	0.41795		3.12131	0.40811		5.41352	0.40328
50	4	11.564	0.41082	4.5	17.1377	0.41628	5	24.3082	0.42236
60		10.819	0.4046		16.0932	0.40834		22.9083	0.41256
70		10.189	0.40107		15.1979	0.40361		21.6985	0.40649
80		9.6296	0.39962		14.4163	0.40082		20.6314	0.40276
90		9.1247	0.3996		13.714	0.39960		19.6706	0.4007
100		8.6721	0.4004		13.0674	0.39960		18.8048	0.39969
